# Impact of digital health management on clinical outcomes during post-PCI outpatient care in patients with acute coronary syndrome: study protocol for a multicentre, randomized controlled trial

**DOI:** 10.3389/fcvm.2025.1555544

**Published:** 2025-05-20

**Authors:** Hang Yu, Wei Zhang, Guoliang Li, Tao Chen, Shaonong Dang, Xiaofeng Ma, XiaoWei Zhang, Xiaofeng Ma, Zhibin Hong, Pengyi He, Xiaohui Xu, Xiuying Chen, Yanyan Geng, Xinjun Lei

**Affiliations:** ^1^ Department of Cardiovascular Medicine, The First Affiliated Hospital of Xi'an Jiaotong University, Xi'an, China; ^2^ Department of Neonatology, The First Affiliated Hospital of Xi’an Jiaotong University, Xi’an, China; ^3^ Key Laboratory of Environment and Genes Related to Diseases, Xi'an Jiaotong University, Ministry of Education, Xi'an, China; ^4^ Key Laboratory for Disease Prevention and Control and Health Promotion of Shaanxi Province, School of Public Health, Global Health Institute, Health Science Center, Xi’an Jiaotong University, Xi’an, China; ^5^ Department of Cardiovascular Medicine, Qinghai Province Cardiovascular and Cerebrovascular Disease Specialist Hospital, Xining, China; ^6^ Department of Cardiovascular Medicine, The Second Hospital & Clinical Medical School, LanZhou University, LanZhou, China; ^7^ Department of Cardiovascular Medicine, Affiliated Nanhua Hospital, Hengyang University of South China, Hengyang, China; ^8^ Department of Cardiovascular Medicine, First People’s Hospital of Tianshui, Tianshui, China; ^9^ Department of Cardiovascular Medicine, The Fifth Affiliated Hospital of Xinjiang Medical University, Urumqi, Xinjiang, China; ^10^ Department of Cardiovascular Medicine, XD Group Hospital, Genertec Universal Medical, Xi’an, China; ^11^ Department of Cardiovascular Medicine, QingHai Provincial People’s Hospital, Xining, Qinghai, China; ^12^ Medical College, Qinghai University, Xining, China

**Keywords:** acute coronary syndrome, percutaneous coronary interventions, digital health management, cardiovascular events, post-PCI outpatient care

## Abstract

**Introduction:**

Coronary heart disease is becoming more common in developing nations. Acute coronary syndrome (ACS) poses a major challenge to global health; however, access to cardiac rehabilitation and preventative measures is limited. There is an urgent need for affordable and widely accessible alternative delivery models to resolve this treatment gap. Research conducted by both local and international experts has focused on the role of smartphones in offering rehabilitation guidance and secondary prevention for patients. Rehabilitative effects have mainly been observed in areas where risk factors and behavioural adjustments can be altered. However, high-quality, evidence-based randomized controlled trials (RCTs) are lacking. Furthermore, the previous studies were single-centre studies with restricted case sources, thereby leading to relatively weak external validity of the research findings. Additionally, most of the previous studies have evaluated cardiac function indicators, with limited assessments of patients' clinical endpoint indicators, quality of life, and psychological aspects.

**Method and analysis:**

The purpose of this study is to explore the development of a smartphone-based systematic and extensible digital management model for post-percutaneous coronary intervention (PCI) outpatient care among ACS patients. We aim to assess whether the integration of a digital management model with conventional postoperative follow-up management is more beneficial for patient recovery during the outpatient rehabilitation process than conventional postoperative follow-up management alone. We propose a single-blind, multicentre RCT of 1,366 ACS patients who underwent PCI and who completed 12 months of follow-up. In the experimental group, within one year after discharge, participants will receive both the current standard postoperative follow-up management and additional management via a digital platform. In the control group, within one year after discharge, participants will receive only the current standard postoperative follow-up management. The primary clinical endpoint of this study is major adverse cardiovascular and cerebrovascular events (MACCEs), whereas the secondary clinical endpoints include cardiac function indicators, quality of life scores, and mental health scores, among other endpoints.

**Conclusions:**

This clinical trial will provide evidence to demonstrate whether digital health management is a better approach for improving clinical outcomes in patients with post-PCI ACS.If proven effective, this digital model could address the global underutilization of cardiac rehabilitation by providing scalable, low-cost solutions, particularly in rural and resource-limited settings. Clinically, it may reduce MACCEs and improve quality of life, while public health systems could integrate this approach to alleviate workforce shortages and geographic barriers.

**Trial Registration Number:**

https://www.clinicaltrial.gov, identifier ChiCTR2400086452.

## Introduction

Acute coronary syndrome (ACS), which is a significant cause of cardiovascular disease-related deaths worldwide, is a sudden heart condition caused by the rupture or erosion of unstable atherosclerotic plaques in the coronary arteries. This event is followed by the formation of new thrombi (1). The frequency of cardiovascular diseases in China is currently increasing on a yearly basis, and cardiovascular disease-related deaths have become one of the major causes of total deaths among both urban and rural residents (2). The use of percutaneous coronary intervention (PCI) has considerably decreased the death rate in patients with ACS (3). However, patients still experience adverse cardiac events such as cardiac insufficiency, myocardial remodelling, recurrent refractory angina, repeat revascularization, and cardiac death (4), which significantly impact the overall prognosis and quality of life of patients with ACS. Therefore, PCI is not a one-time solution, and numerous issues remain to be addressed after PCI. Consequently, effective cardiac rehabilitation and secondary prevention (CR/SP) for patients after PCI are particularly important.

Currently, multiple studies (5, 6) have confirmed that standardized CR/SP for patients who undergo PCI can effectively control risk factors for cardiovascular diseases, enhance adherence to cardioprotective medications, improve patients' quality of life, and enhance postcardiovascular event prognosis. Specifically, this approach can reduce cardiovascular mortality by 26% and hospitalization rates by 18%. Mampuya Wamer M (7) proposed that cardiac rehabilitation can decrease mortality, alleviate symptoms, promote smoking cessation and alcohol restriction, improve exercise tolerance, and control risk factors; however, they also noted that the effectiveness of cardiac rehabilitation is limited by issues such as referral problems and poor registration. Lv et al. (8) implemented continued management for PCI patients through an out-of-hospital PCI club, which improved patients' adherence to medical advice, effectively controlled the risk factors for coronary heart disease, reduced cardiovascular events, and facilitated postoperative recovery. Sora Baek et al. (9) conducted educational courses and cardiac rehabilitation training for patients residing in rural areas within the community. The results revealed significant reductions in patient weight and blood glucose levels, as well as improvements in the 6-minute walk test (6MWT) results, thereby confirming the effectiveness of this community-based cardiac rehabilitation management model. Thus, standardized management of cardiac rehabilitation for patients who undergo PCI is particularly important, as this management approach can improve patients' cardiac rehabilitation levels and further enhance their quality of life.

However, CR/SP services are still not fully utilized on a global scale, despite the recognized benefits. Therefore, innovative strategies are needed to implement evidence-based treatments. The CR/SP model should be accessible, affordable, and scalable to effectively treat coronary heart disease and resolve the gap between existing practices and recommended guidelines (10). With the rapid advancement of the internet and the growing variety of communication techniques, smartphone-based programs have demonstrated effectiveness as being alternative methods for delivering rehabilitation and secondary prevention to patients with coronary heart disease; this is especially true for the alteration of risk factors and changes in behaviour. In China, the rapid growth of smartphone and social media users has led to the formation of an effective platform for offering CR/SP services through these media. To date, research reports from both domestic and international sources have examined the application of smartphones for providing rehabilitation guidance and secondary prevention for patients (11, 12). Notably, implementation studies in Europe have demonstrated the clinical viability of mHealth platforms for coordinating secondary prevention strategies in post-myocardial infarction populations, particularly through structured remote follow-up systems (13). Mobile apps technology can be used as a safe choice to improve the health status and enhance physical vitality of elderly patients with coronary heart disease (14). In the study by Effie et al. (15), an internet- and mobile phone-based management model was found to effectively improve patients' cardiac rehabilitation outcomes and enhance their adherence. With particular respect to patients in rural areas, this internet-based management model provides considerable convenience, thus effectively addressing the issue of inaccessible medical care due to their remote locations. In the study by Pemille Lunde (16), the significant role of mobile applications in patients' cardiac exercise rehabilitation and the control of cardiovascular disease risk factors was fully demonstrated. Tashi Dorje et al. (11) used the SMART-CR/SP system via smartphone WeChat software to provide at-home cardiac rehabilitation and secondary prevention initiatives for post-PCI coronary heart disease patients. The results showed that the use of the SMART-CR/SP system could significantly improve patients' post-PCI cardiac functional capacity, blood pressure control, medication adherence, and knowledge regarding lipids and coronary heart disease. Mobile applications, which have the advantages of high accessibility, opportunities for two-way communication, and the ability to exchange information in real-time, serve as significant assets for disease prevention and management methods, including mass public health education, personalized healthcare, and customized treatment.

When considering the somewhat restricted number of clinical studies focusing on the success of internet- and smartphone-based outpatient rehabilitation management for post-PCI ACS patients, as well as the lack of large-sample, multicentre RCTs, the case sources are somewhat restricted, and the generalizability of the research findings is weak.

Therefore, the aim of this study is to conduct a multicentre RCT in China. Additionally, previous clinical assessments have primarily focused on patients' cardiac function indicators, whereby they often used the 6-minute walk distance as the main outcome measure, with limited research on clinical endpoint indicators such as major adverse cardiovascular and cerebrovascular events (MACCEs), patients' quality of life, mental health, and other cardiac function indicators (such as brain natriuretic peptide). Therefore, in this study, in addition to focusing on the 6-minute walk distance as a cardiac function indicator, we will also assess MACCEs, quality of life, and other cardiac function evaluation indicators within and between groups to better understand the impacts of digital health management on the outpatient rehabilitation effect for ACS patients who undergo PCI. These findings will provide a basis for an accessible, affordable, and scalable CR/SP model and provide the foundation for improvements in home-based rehabilitation management.

## Methods and analysis

### Study hypotheses

The purpose of this trial is to test the hypothesis that a digital health management model that combines smartphone applications with the current routine postoperative follow-up management model is superior to the current routine postoperative follow-up management model alone for the outpatient rehabilitation of patients with ACS who underwent PCI.

### Study design

**Figure 1:**
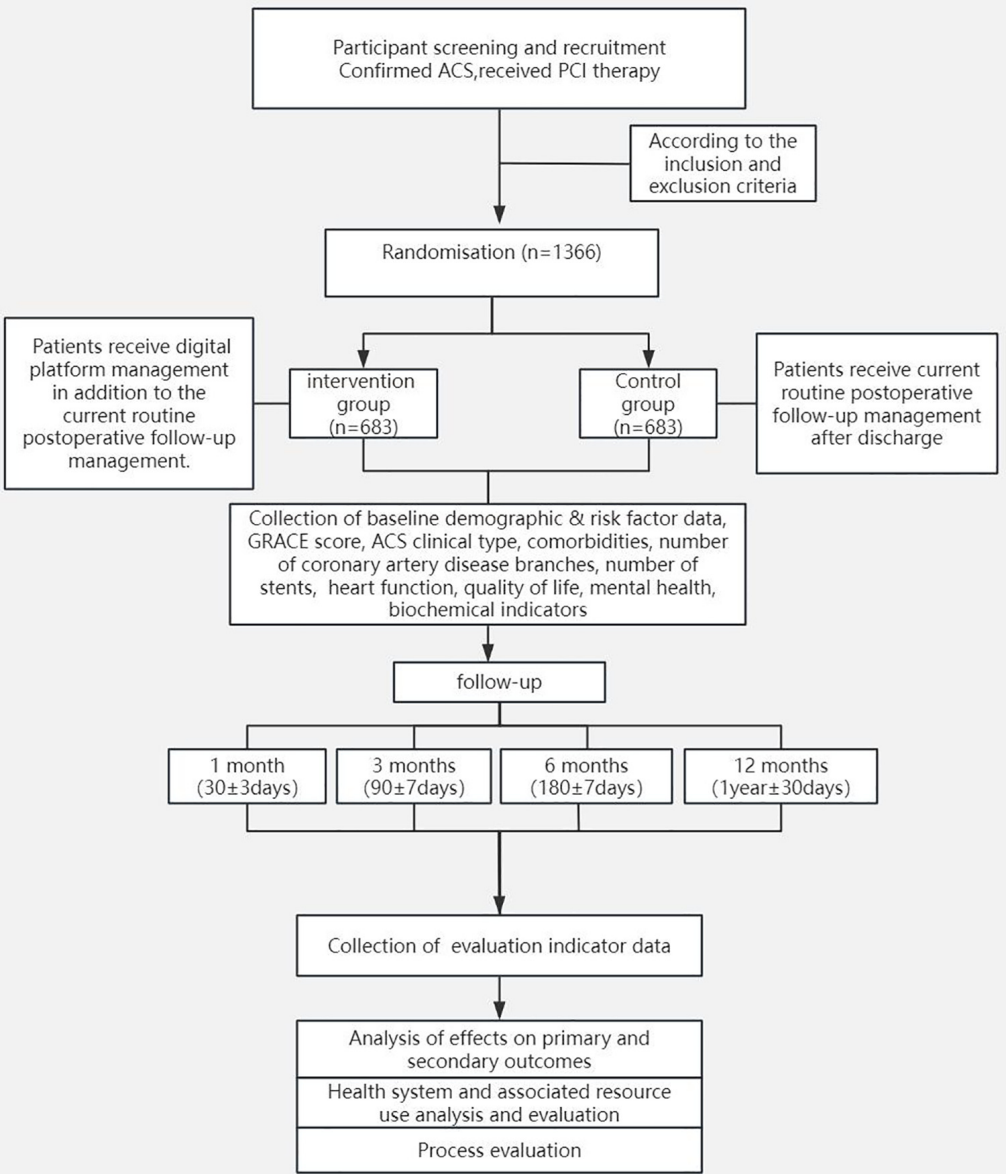
Flowchart of the clinical trial design.

This study will be a multicentre, open-label, endpoint-blinded RCT. The trial will be conducted at 9 medical centres in China and will include 1,366 patients. The flowchart of the study process is shown in Figure 1.

This clinical trial will adhere to the principles outlined in the Declaration of Helsinki. The Clinical Trial Ethics Committee of The First Affiliated Hospital of Xi'an Jiaotong University, as well as each medical centre, reviewed and approved the clinical trial protocol and informed consent documents. All of the patients participating in the study must provide written informed consent. The study protocol is registered with the China Clinical Trial Registry (ChiCTR2400086452).

### Study population and randomization

A total of 1,366 ACS patients who underwent PCI and who meet the inclusion criteria without meeting any exclusion criteria will be included from nine research centres. The allocation of patients will be randomized via an interactive web randomization system.

### Inclusion criteria

a. Participants aged between 18 and 70 years;
b. Newly diagnosed patients meeting the clinical diagnostic criteria for ACS (17);
c. Individuals who have undergone PCI and subsequently met the criteria for hospital discharge;
d. Patients with an anticipated survival duration exceeding one year;
e. Patients who possess adequate learning, comprehension, and communication abilities, thus enabling them to grasp the pertinent aspects of the trial and actively collaborate with research requirements;
f. Participants who are proficient in smartphone usage, capable of operating specialized “Jiuyi” software, and expressing interest in participation;
g. Patients with comprehensive clinical records who voluntarily elect to participate, have obtained familial consent, and are amenable to signing the informed consent form.

### Exclusion criteria

a. Individuals presenting with severe cardiac, hepatic, or renal dysfunction, immune system disorders, or any other acute or chronic severe systemic illnesses;
b. Patients who suffer severe complications, including dissection, shock, or substantial haematoma, after PCI;
c. Patients diagnosed with haemorrhagic diseases or malignant tumours;
d. Patients with untreated ventricular tachycardia, advanced heart failure, intractable hypertension, or hypotension;
e. Individuals with a prior history of cardiac surgical intervention;
f. Patients affected by mental illness, profound cognitive impairments, or an absence of autonomous behavioural capacity;
g. Participants unable to use a smartphone for the duration of the trial due to visual, auditory, or motor impairments;
h. Residents in locations lacking internet access;
i. Patients declining to provide written informed consent.

### Study procedures

This study will adopt a stratified block randomization approach.

(1) First, patients will be stratified based on their GRACE scores.

For GRACE scores, with respect to the postdischarge patients, the low-risk category will consist of patients with scores up to 88, the moderate-risk category will consist of those scoring between 89 and 118, and the high-risk category will consist of patients scoring greater than 118. Patients in the low-risk category will have a score of 108 or less, those in the moderate-risk category will have scores ranging from 109–140, and patients in the high-risk category will have scores greater than 140.

Randomization will be stratified by GRACE risk score (low, moderate, high) and center. Blocks of 4 patients within each stratum will be randomized 1:1 using a web-based system (Sealed Envelope™), ensuring allocation concealment.
(2) Block randomization within strata

Randomization within strata will be achieved via block randomization as follows.

(1) Numbering: Patients will be numbered on the basis of the order of their enrolment within each stratum. For example, the first high-risk patient will be labelled H001, the first moderate-risk patient will be labelled M001, and the first low-risk patient will be labelled L001, thereby generating continuous ID numbers within each stratum.
(2) Block formation: Every four consecutively enrolled patients within each stratum will be grouped into one block; thus, each block will include four patients.
(3) Randomization: A seed number will be established, and four random numbers corresponding to the IDs will be generated within each block. The four subjects within each block will then be ranked according to the random numbers. The top two subjects in each block will be assigned to the experimental group, whereas the bottom two subjects will be assigned to the control group. The allocation results will be sorted in ascending order by ID number to form a randomization table within each stratum.

(3) Patient grouping

Researchers at each centre will obtain randomization information for patients from a designated person based on the randomization table and the order of patient enrolment. The participants will be equally divided between the experimental group and the control group.

Experimental Group: Within one year after discharge, patients will receive digital platform management in addition to the current standard postoperative follow-up management.

Control Group: Within one year after discharge, patients will receive the current standard postoperative follow-up management.

## Study endpoints

### Primary endpoint

The primary endpoint of this study is the incidence rate of MACCEs within one year after patient discharge from the hospital.

MACCEs encompass the following events:

(1) All-cause mortality
(2) Cardiac-related mortality
(3) Nonfatal myocardial infarction
(4) Nonfatal stroke
(5) Repeated revascularization procedures

### Secondary endpoints

(1) Incidence rate of all-cause mortality;
(2) Incidence rate of cardiac-related mortality;
(3) Incidence rate of nonfatal myocardial infarction;
(4) Incidence rate of nonfatal stroke;
(5) Incidence rate of repeat revascularization procedures;
(6) Cardiac function indicators, including LVEF, LVESD, LVEDD, 6-minute walk test (6MWT), and brain natriuretic peptide (BNP) levels;
(7) Quality of life scores;
(8) Mental health scores, including anxiety scores and depression scores;
(9) Biochemical indicators.

### Medications

All of the enrolled patients will receive dual antiplatelet therapy for at least 12 months, according to local practices and current guidelines. Patients will be instructed to take a loading dose of aspirin (300 mg) and either clopidogrel (600 mg) or ticagrelor (180 mg) at least 6 h prior to PCI. Heparin or other antithrombotic agents, such as bivalirudin, are used during PCI. After PCI, long-term treatment with 100 mg of aspirin per day is recommended. Treatment with 75 mg clopidogrel daily (or 90 mg ticagrelor twice daily) should be continued for at least 12 months.

### Digital platform (“Jiuyi” Health App)

The digital health management platform employed in this study was implemented through a smartphone application named JiuYi Health App. Patients could register and access the platform by scanning a designated QR code. Data are encrypted and stored on HIPAA-compliant servers.

The app comprises five core functional modules: Health Data Entry, Physician-Patient Communication, Health Guidance, Alert System, and Health Education. Key features are detailed in the Table 1.

**Table 1:** Key features of the digital platform.

Module	Functional Specifications
Health Data Entry	- Patients upload daily health metrics (e.g., blood pressure, glucose levels, medication adherence, exercise duration, dietary patterns, sleep quality, weight, smoking status, blood oxygen saturation, and psychological status) for physician review. - Automated reminders prompt patients to complete missing entries.
Physician-Patient Communication	- Enables virtual consultations and real-time messaging. - Physicians proactively inquire about health status to enhance treatment adherence.
Alert System	- Triggers automated alerts to patients and physicians upon detecting abnormal values. - Sends reminders for scheduled follow-ups.
Health Guidance	- Physicians analyze uploaded data to generate personalized behavioral recommendations. - Identifies and rectifies incomplete data submissions.
Health Education	- Provides disease-specific multimedia educational resources (text, images, videos) to improve health literacy.

### Follow-up

The enrolling centre will conduct clinical follow-ups via either office visits or phone calls at 30 days (±3 days), 3 months (±7 days), 6 months (±7 days), and 12 months (±30 days) to assess outcomes after discharge.

Standard care includes monthly clinic visits for physical exams, medication adjustment, and 6MWT assessments, plus quarterly phone calls to review symptoms and adherence. All patients receive printed guidelines on lifestyle modifications and medications.

### Intervention protocols

#### Control group

Patients received standard post-PCI outpatient care for 12 months, including:

Pre-discharge education on medication adherence, risk factor management, and follow-up schedules.

Structured follow-ups at Months 1, 3, 6, 9, and 12 with the Table 2.

**Table 2:** Standard postoperative follow-up.

Measures	Content
Outpatient monitoring	✓ Routine monitoring: blood pressure, blood glucose, blood lipids, liver function, kidney function, brain natriuretic peptide; medication adherence ✓ Electrocardiogram monitoring: myocardial ischemia, arrhythmia ✓ Medium risk and high-risk patients who have the conditions to wear electrocardiogram monitoring devices for electrocardiogram monitoring can wear electrocardiogram detection devices for monitoring according to their situation after discharge. Monitor electrocardiogram at least once a day within one month after discharge, at least twice a week within three months, and at least once a week within six months. After one year, the monitoring frequency can be adjusted according to one’s own symptoms and follow-up status; If there is discomfort such as chest pain, tightness, palpitations, etc., immediately use wearable devices for electrocardiogram monitoring. ✓ Those who do not have the conditions to wear electrocardiogram testing equipment should regularly go to the hospital to receive electrocardiogram or dynamic electrocardiogram monitoring and evaluation.
Non pharmacological measures	✓ Cardiac rehabilitation therapy should be mainly based on reasonable exercise, while paying attention to a reasonable diet, controlling total calories, and reducing intake of saturated fatty acids, trans fatty acids, and cholesterol. ✓ Weight control: Overweight and obese patients lose 5%–10% weight within 6–12 months. Target value: 18.5 ≤ Body mass index ≤ 25 kg/m^2^; Waist circumference should be controlled within 90 cm for males and 85 cm for females. Weight loss can be achieved by reducing energy intake and exercising. ✓ Do not smoke: Strictly quit smoking, including passive smoking. ✓ Limit alcohol consumption: Strictly control alcohol intake (≤20 g/d for males and ≤10 g/d for non-pregnant females), with a recommended daily alcohol intake of <100 grams/week or <15 grams/day. ✓ Diet: A healthy and balanced diet. Suggest increasing the intake of vegetables, fruits, and whole grains. ✓ Psychological adjustment: Provide patients with multiple, patient and procedural education to fully understand their illness and severity, relieve tension, improve follow-up and treatment compliance, and strengthen self-management; It is necessary to identify the psychological problems of patients and provide targeted treatment. ✓ Exercise: Encourage physical activity and recommend 30–60 min of moderate intensity aerobic exercise per day. However, corresponding exercise guidance should also be developed for patients based on the results of myocardial ischemia and heart function assessment before discharge. In principle, all ACS patients during the rehabilitation period should gradually increase their exercise intensity within the range of not inducing myocardial ischemia and heart function tolerance. Regular reevaluation should be conducted through exercise electrocardiogram or cardiopulmonary function measurement, 6 min walking distance, and dynamic electrocardiogram, and exercise intensity adjustment should be guided.
Pharmacological measures	✓ Strictly follow the doctor's advice when taking medication. If there are special requirements for certain medications (such as long-term use, dosage, etc.), explain them clearly to the patient. ✓ Blood lipids: All recent ACS patients (within 1 year of onset) can be classified as a high-risk population for ASCVD. The target value for lipid-lowering therapy is LDL-C < 1.4 mmol/L (55 mg/dl) and a decrease of ≥50% from baseline. ✓ Blood pressure: Control blood pressure to <140/90 mm Hg, and if tolerable, it can be reduced to <130/80 mm Hg. ✓ Blood sugar: General population: HbA1c ≤ 7%, For patients with high risk of hypoglycemia, such as older patients, longer course of diabetes, impaired renal or liver function, it can be relaxed to <7.5% or < 8.0%.
Rehabilitation therapy	ACS patients should actively participate in cardiac rehabilitation therapy.

#### Experimental group intervention

Within one year after discharge, patients in the experimental group will receive digital platform management based on current routine outpatient postoperative follow-up management.

Specific requirements: In addition to completing all the tasks of the control group, patients in the experimental group are also required to upload daily health data through the platform after discharge, including blood pressure, blood sugar, exercise, diet, sleep, medication information, discomfort symptoms, mental health status, smoking, and other data as required. By uploading data, doctors can timely understand the patient's disease situation and provide personalized rehabilitation guidance, enabling patients to regulate their daily behavior outside the hospital and actively participate in the disease rehabilitation process.

The period for patients to use the digital platform is within 12 months after discharge, and patients need to have follow-up visits at 1, 3, 6, 9, and 12 months after discharge.

Doctors will provide guidance and analysis on the health data uploaded by patients every week, and form a health guidance report for feedback to patients. If communication is needed, patients can be directly communicated through the platform's doctor-patient interaction function to guide their condition and recovery. If patients have any questions or doubts and need to consult doctors, they can also directly communicate through the platform.

#### Follow up matters

The time points for patient follow-up in the study were 1, 3, 6, 9, and 12 months after discharge, totaling 5 times. The specific follow-up contents are shown in the Table 3 below.

**Table 3:** The time points and content for patient follow-up in the study.

Follow up frequency	Follow-up time window	Content	Data
The first time	1 month ± 3 days after discharge	Complete blood routine, biochemistry, myocardial enzymes BNP, Electrocardiogram, cardiac ultrasound and other examinations; Complete the assessment of quality of life questionnaire and mental health questionnaire; For patients with multi vessel, complex, and CTO lesions, if there are chest pain symptoms, hospitalization for coronary angiography is required.	Main indicators: The incidence of major adverse cardiovascular and cerebrovascular events (MACCE); Secondary indicators: The incidence rates of various events in MACCE; Cardiac function indicators; Quality of life rating; Psychological health score; Blood and biochemical indicators Safety indicators: bleeding events, etc
The second time	Within 3 months ± 7 days after discharge		
The third time	6 months ± 7 days after discharge		
The 4th time	9 months ± 7 days after discharge		
The 5th time	12 months ± 30 days after discharge		

The period for patients to use the digital platform is within 12 months after discharge, and patients need to have follow-up visits at 1, 3, 6, 9, and 12 months after discharge (Table 4).

**Table 4:** Follow-up schedule.

Timepoint	Assessments
Baseline (Discharge)	Demographics, GRACE score, LVEF(echocardiography), BNP, lipid profile, 6MWT
1 Month (±3 days):	Clinic visit: Medication adherence (pill count), adverse events, 6MWT. Lab tests: BNP, renal function.
3 Months (±7 days)	Clinic visit: LVEF (repeat echocardiography), quality of life (SF-36 questionnaire), mental health (HADS score).
6 Months (±7 days)	Phone call: Symptom assessment, MACCE screening. Lab tests: Lipid profile, HbA1c.
12 Months (±30 days):	Clinic visit: Final LVEF, 6MWT, SF-36, HADS, MACCE adjudication.

Doctors will provide guidance and analysis on the health data uploaded by patients every week, and form a health guidance report for feedback to patients. If communication is needed, patients can be directly communicated through the platform's doctor-patient interaction function to guide their condition and recovery. If patients have any questions or doubts and need to consult doctors, they can also directly communicate through the platform.

#### Follow up matters

The time points for patient follow-up in the study were 1, 3, 6, 9, and 12 months after discharge, totaling 5 times. The specific follow-up contents are shown in the Table 5 below.

**Table 5:** A timeline for study follow-up.

No.	Category	Item	Specific Indicator	Baseline	Follow-up Periods			
					FU1	FU2	FU3	FU4
					30 ± 3days	90 ± 7days	180 ± 7days	365 ± 30days
1		Informed Consent		×				
2		Inclusion/Exclusion		×				
3		Group Assignment		×				
4	Baseline Data	Demographic Information		×				
		Disease Characteristics		×				
5	Clinical evaluation indicators	MACCE	All-cause mortality		×	×	×	×
			Cardiac mortality		×	×	×	×
			Non-fatal myocardial infarction		×	×	×	×
			Non-fatal stroke		×	×	×	×
			Repeat revascularization		×	×	×	×
		Cardiac Function Parameters	LVEF	×	×	×	×	×
			LVESD	×	×	×	×	×
			LVEDD	×	×	×	×	×
			6MWT	×	×	×	×	×
			BNP	×	×	×	×	×
		Quality of Life		×	×	×	×	×
		Mental Health Assessment	Anxiety score	×	×	×	×	×
			Depression score	×	×	×	×	×
		Biochemical Parameters		×	×	×	×	×
6		Clinical routine examination and evaluation		×	×	×	×	×
7		Deviations			×	×	×	×
8		Adverse Events			×	×	×	×

## Data collection and management

Researchers will receive thorough training on the research requirements to improve data quality and will be involved in the data collection and entry procedures. Data that are required for this clinical trial, such as baseline clinical characteristics, medical treatments, laboratory results, interventional treatments and outcomes, will be collected by investigators at each medical centre. An independent data monitoring committee has been established and will review the accuracy of the data on a quarterly basis.

## Trial status

This clinical trial is still in progress, with participants being recruited from nine medical centres in China. The number of subjects targeted at each medical centre will depend on the daily coronary angiography patient count and the requests from each centre.

## Sample size and statistical analyses

This study will adopt a multicentre, open-label, RCT design, with the primary endpoint being MACCEs at one year after patient discharge. The sample size will be calculated based on this primary endpoint.

The following sample size calculation formula was used:

$$n=\frac{2\bar{p}\bar{q}{\left(Z_{\alpha }+Z_{\beta }\right)}^{2}}{{\left(p1-p2\right)}^{2}}$$

where “*n*” represents the sample size per group, and Z*α* and Z*β* are obtained from statistical tables. When *α* = 0.05, Z_0.05_ = 1.96. For a power (test efficacy) of 0.9, Z*β* = 1.28; for a power of 0.8, *β* = 0.2, and Z*β* = 0.84. In this study, *α* = 0.05 and *β* = 0.2 were chosen; thus, Z*α* and Z*β* are 1.96 and 0.84, respectively.

“p^−^” represents the mean of p1 and p2, whereas “q^−^” represents the mean of 1-p1 and 1-p2. Additionally, p1 and p2 are the event rates for the experimental and control groups, respectively. Based on previous research (18) and clinical considerations, the one-year MACCE rate for the control group was set at 20% (p2 = 20%). After intervention, the MACCE rate in the experimental group is expected to be reduced by 30%, thus resulting in p1 = 14%.

Using these values in the formula, the required sample size per group (n) is calculated as 615, thus yielding a total sample size of 1,230. With a potential 10% dropout rate considered, the necessary total sample size for this study is 1,366, with 683 patients being allocated per group.

### Statistical analysis

This study will use two-sided tests for the statistical analysis, with a significance threshold set at *α* = 0.05. A *p* value less than 0.05 will be regarded as being statistically significant. The principles for data description and hypothesis testing are as follows:

(1) Statistical description

An analysis will be conducted on the continuous variables to ascertain if they exhibit a normal distribution. Continuous variables that follow a normal distribution will be presented as the means with standard deviations, whereas those that do not follow a normal distribution will be shown as medians with interquartile ranges.

Categorical variables will be presented as frequencies (number of cases) and percentages.

(2) Statistical inference

For comparisons between the groups, normally distributed continuous variables will be analysed via independent sample *t* tests. For nonnormally distributed continuous variables, comparisons will be performed via the Mann–Whitney *U* test. Within-group comparisons involving continuous variables with a normal distribution will utilize paired *t* tests, whereas those with nonnormal distributions will employ Wilcoxon rank-sum tests.

*Χ*^2^ tests, Fisher's exact tests, or nonparametric rank-sum tests will be used to compare the categorical variables.

Further analyses will include multivariate model adjustments to account for potential confounding factors and to determine the stability of the results. For comparisons of data at different time points, repeated-measures ANOVA will be used to assess between-group differences over time. Kaplan–Meier curves will be plotted to illustrate the cumulative occurrence of cardiovascular events over time, and log-rank tests will be used for comparisons. Additionally, baseline characteristics will be compared between participants who are included and not included in the final analysis to verify if the missing data are random. Cox proportional hazards models will be used to estimate the hazard ratio (HR) and 95% confidence interval (CI) for the primary endpoint.

## Discussion

This study will evaluate the development of a smartphone-based systematic and extensible digital management model for post-PCI outpatient care among ACS patients. Specifically, the aim of this study is to assess whether the integration of a digital management model with conventional postoperative follow-up management is more beneficial for patient recovery during the outpatient rehabilitation process than conventional postoperative follow-up management alone. To our knowledge, no prior studies have investigated the effectiveness of a CR/SP service delivery model using smartphones and social media.

Standardized management of cardiac rehabilitation for patients after PCI is crucial, as it can help to enhance the level of cardiac rehabilitation and further improve patients' quality of life (19). However, despite its recognized benefits, CR/SP services remain underutilized throughout the world (20). Statistics from countries such as the United States and Australia show that patient participation rates for these services vary from 30%–45%, with dropout rates of approximately 40%–55% being observed (21). Access to CR/SP services remains limited in low- and middle-income countries, with less than 25% of these countries offering such programs. Although there have been improvements in the medical management of coronary heart disease in China in recent years, CR/SP programs (which are currently conducted in outpatient settings) experience certain access barriers and are thereby not fully utilized (22). Consequently, innovative strategies are needed to implement evidence-based treatments. CR/SP models must be easily accessible, economical and scalable to offer this effective therapy, thus bridging the divide between current practices and guideline recommendations for coronary heart disease patients in China.

Compared with conventional telephone and SMS assistance, interventions via the internet and smartphones exhibit a potentially more effective and flexible platform for disease management, particularly in modifying risk factors and altering behaviour (23, 24). Access to tertiary and secondary prevention healthcare is often constrained in China and other LMICs. These advanced technological features may significantly enhance the provision of essential elements of contemporary CR/SP to numerous CHD patients who may not have otherwise accessed these crucial services. Due to advantages such as high accessibility, opportunities for two-way communication, and the capability of mobile applications to instantly share information, they provide effective tools for disease prevention and management, thus incorporating widespread public health education, personalized treatment, and individualized care.

There are some limitations that need to be noted. First, the requirement for smartphone proficiency may exclude older or less tech-literate populations, limiting generalizability. Second, internet dependency could hinder implementation in regions with poor connectivity. However, the modular design of the platform allows offline functionality (e.g., preloaded educational content), which may mitigate this barrier. Future studies should test adaptations for low-resource settings, such as SMS-based interventions or community health worker-supported models.

## Conclusion

This multicentre RCT aims to evaluate the effectiveness of a self-developed smartphone application featuring functions such as patient health data uploading and feedback, doctor-patient interaction, early warning alerts, health guidance, and disease-related knowledge learning for the daily management of patient health, in addition to routine follow-up care. The objective of this study is to assess the efficacy of this smartphone-based specialized software in providing home-based cardiac rehabilitation and secondary prevention for patients who underwent PCI. The aim of this research is to provide evidence to support an accessible, affordable, and scalable model of cardiac rehabilitation and secondary prevention, thereby providing a foundation for improving home-based rehabilitation management.

If the digital model proves superior to standard care, it could be integrated into national guidelines for post-PCI management. Clinicians may adopt it to extend rehabilitation access to underserved populations, while policymakers could subsidize smartphone-based programs to reduce disparities. For patients, this approach may empower self-management, reduce MACCE risks, and lower healthcare costs through preventive care.
